# Dietary Karaya Saponin and *Rhodobacter capsulatus* Exert Hypocholesterolemic Effects by Suppression of Hepatic Cholesterol Synthesis and Promotion of Bile Acid Synthesis in Laying Hens

**DOI:** 10.1155/2010/272731

**Published:** 2010-06-30

**Authors:** Sadia Afrose, Md. Sharoare Hossain, Ummay Salma, Abdul Gaffar Miah, Hirotada Tsujii

**Affiliations:** Laboratory of Animal Biotechnology, Interdisciplinary Graduate School of Science and Technology, Shinshu University, Minamiminowa-mura, Nagano 399-4598, Japan

## Abstract

This study was conducted to elucidate the mechanism underlying the hypolipidemic action of karaya saponin or *Rhodobacter (R.) capsulatus*. A total of 40 laying hens (20-week-old) were assigned into four dietary treatment groups and fed a basal diet (as a control) or basal diets supplemented with either karaya saponin, *R. capsulatus*, or both for 60 days. The level of serum low-density-lipoprotein cholesterol and the levels of cholesterol and triglycerides in the serum, liver, and egg yolk were reduced by all the supplementations (*P* < .05). Liver bile acid concentration and fecal concentrations of cholesterol, triacylglycerol, and bile acid were simultaneously increased by the supplementation of karaya saponin, *R. capsulatus*, and the combination of karaya saponin and *R. capsulatus* (*P* < .05). The supplementation of karaya saponin, *R. capsulatus*, and the combination of karaya saponin and *R. capsulatus* suppressed the incorporation of ^14^C from 1-^14^C-palmitic acid into the fractions of total lipids, phospholipids, triacylglycerol, and cholesterol in the liver in vitro (*P* < .05). These findings suggest that the hypocholesterolemic effects of karaya saponin and *R. capsulatus* are caused by the suppression of the cholesterol synthesis and the promotion of cholesterol catabolism in the liver.

## 1. Introduction

Saponins are natural detergent forms of a heterogeneous group of triterpene or steroid glycosides that occur in many hundreds of plant species [1]. A number of studies have shown that different kinds of saponins lower serum cholesterol levels in a variety of animals and human subjects [2–4]. Recently, we have reported that karaya saponin is the best hypocholesterolemic substrate, because karaya saponin reduced serum cholesterol concentration by 34% in rats while tea, quillaja, or soyabean saponins resulted in reductions of less than 20% [5]. We have also reported that *Rhodobacter capsulatus *(*R*. *capsulatus*), which is a photosynthetic bacterium, exhibits hypocholesterolemic activities in laying hens (15%) [6], Japanese quails [7], rats [8], and pigs [9]. Furthermore, we found that the combination of karaya saponin and *R. capsulatus *as supplementation in a hen's diet causes much larger reductions in serum (32%) and egg yolk cholesterol (18%) levels than saponin alone [10]. However, the mechanism underlying the hypocholesterolemic effect of karaya saponin and *R. capsulatus *has not been clarified.

 Recent studies have revealed that bile acids are ligands of several nuclear hormone receptors involved in regulating bile acid synthesis, transport, and cholesterol metabolism. The cholesterol pool is derived from two major sources: the synthesis of cholesterol by the liver and the absorption of cholesterol from the intestine [11]. The cholesterol pool rarely changes much because cholesterol input is approximately balanced by cholesterol output via factors such as excretion in bile/feces, skin excretion, and steroid hormone synthesis [12]. Suppression of cholesterol synthesis is also associated with hypocholesterolemia. Bile acid synthetic pathway is the major pathway of cholesterol catabolism. Zhao et al. [13] suggested that saponin lowers serum cholesterol by increasing the conversion of cholesterol into bile acids through the upregulation of a rate-limiting enzyme, CYP7A1, in hepatic bile acid synthesis. Therefore, it is possible that dietary karaya saponin and *R. capsulatus *exert hypocholesterolemic activity by the suppression of hepatic cholesterol synthesis and/or the promotion of bile acid synthesis in laying hens. 

 Thus, in the present study, we investigated the effect of karaya saponin and *R. capsulatus *on lipid metabolism in laying hens.

## 2. Materials and Methods

### 2.1. Birds, Management and Diets

 A total of 40 Boris Brown ready-to-lay pullets with almost the same live weight were supplied by a local commercial flock and reared in individual cages (30 × 40 cm). They were reared in accordance with the “Guidelines for Regulation of Animal Experimentation, Faculty of Agriculture, Shinshu University” and this study was approved by an ethics committee. The birds were fed a balanced commercial layer diet (Toyosashi Shiryo, Kabushiki Gaisha, Aichi, Japan) and reared as previously described in [6]. The basal diet contained (in percentages): ground yellow corn, 56; soybean meal, 14.5; sesame meal, 7; corn gluten meal, 2.5; rice polish, 8; fish meal, 1; calcium carbonate, 7.68; dicalcium phosphate, 1.32; common salt, 0.45; animal fat, 1; and vitamin premix, 0.25. The calculated [14] nutrient composition of the basal diet was 2,935 kcal/kg of ME, 16.46% CP, 2.9% Ca, 0.34% available P, 0.54% Met + Cys, 0.71% Lys, and 86 *μ*g/g of cholesterol. There is no difference in fatty acid composition in experimental diets. Hens were assigned into four dietary treatment groups and fed the basal diet or basal diets supplemented with either *R. capsulatus *(0.04%), karaya saponin (75 mg/kg diet), or both for a 60-day feeding period. Karaya saponin was obtained from Nacalai Tesque, Kyoto, Japan. *R*. *capsulatus *cells were grown in outdoor culture under natural illumination as previously described in [15]. The diets and water were provided ad libitum throughout the experiment, and a photoperiod of 16L : 8D was used.

### 2.2. Sample Preparation and Enzymatic Analysis

 The laying hens were weighed individually prior to blood collection at the beginning and at the end of each 2-week feeding period. Blood was collected from the brachial wing vein using sterilized syringes and needles. After 1 h standing at room temperature, serum was isolated by centrifugation at 1150 × g for 10 min. Serum samples were stored at −80°C until analysis. During the 60-day feeding period, feces were collected weekly from the control and treatment groups of laying hens. Cholesterol in the serum, egg yolk, liver, and feces were measured spectrophotometrically (APEL Co., Saitama, Japan) using a commercial kit (Wako Cholesterol E, Wako Pure Chemical Industries Ltd., Tokyo, Japan). Triacylglycerides were measured using a commercial kit (Wako TG, Wako Pure Chemical Industries Ltd., Tokyo, Japan). High-density-lipoprotein (HDL) cholesterol in the serum was determined enzymatically using a commercial kit (Wako HDL-cholesterol, Wako Pure Chemical Industries Ltd.). Low-density-lipoprotein (LDL) cholesterol was determined using a commercial kit (Daiichi Pure Chemicals Co. Ltd, Tokyo, Japan) as described previously in [16]. For fecal and hepatic bile acid determination, feces and liver samples were ground and lyophilized, and 50 mg of the lyophilized sample was placed in a centrifuge tube containing a 1 ml mixture of *t*-butanol and water (1 : 1, v/v) [17]. The tubes were vigorously vortexed, incubated for 20 min in a gently shaken water bath at 37°C and centrifuged at 1,000 × g for 2 min. The supernatant was decanted into another tube and the bile acids were measured spectrophotometrically using a commercial kit (Wako TBA, Wako Pure Chemical Industries Ltd.).

### 2.3. Fatty Acid Determination

 Total lipid extracts of yolk samples were transmethylated into fatty acid methyl esters and separated using a gas chromatograph (Shimadzu, GC14B, Kyoto, Japan) and an Omegawax 250 capillary column (30 m × 0.25 mm i.d.; 0.25 *μ*m thickness; Supelco, Bellefonte, PA, USA) with cyanopropyl methyl silicone as stationary phase. Helium was used as the carrier gas at a constant flow rate of 4.7 ml/min. The following oven temperature program was used: 100°C held for 1 min, increased to 160°C at 40°C/min then to 240°C at 7°C/min, and 240°C held for 10 min. Peaks were separated using a flame-ionization detector and quantified with an electric integrator (Shimadzu, CR-1A, Kyoto, Japan) using pure standard mixtures (Sigma, St. Louis, 151 MO, USA). We adopted the weight percentage of each fatty acid in all detected fatty acids as a measurement value.

### 2.4. Incorporation of 1-^14^C-Palmitic Acid into Liver Tissue

 At the end of the feeding period, the laying hens were decapitated, and left liver lobes were collected and chilled in ice-cold phosphate buffer solution (PBS). Liver lobes were sliced into 1 mm pieces, lightly pressed on sterilized paper and weighed prior to use in incubations with 1-^14^C-palmitate. About 0.5 g of liver slice was placed in the tube with 990 *μ*l of PBS and 37 KBq (10 *μ*l) of 1-^14^C-palmitic acid (specific activity: 1.92 GBq/mmol; American Radiolabeled Chemicals Inc., St. Louis, MO, USA). The tubes were kept closed during the incubation period and agitated in a water-bath shaker at 39°C for 3 hours. At the end of the incubation, the tubes were placed in a freezer at −40°C to stop the reaction. Tissue and medium were transferred to a cup containing five times their volume of chloroform-methanol 2 : l and homogenized. The homogenate was filtered through cotton filter paper. The residue was homogenized again with five times its volume of chloroform-methanol 2 : 1. This extraction step was repeated five times and the combined extracts were centrifuged at 1,150 × g for 10 min. The chloroform layer was washed with saline solution three times in accordance with Folch et al. [18], which removed 99% of the water-soluble radioactive materials and then evaporated to dryness. Aliquots of hepatic lipids were separated by thin-layer chromatography (TLC) using Silica Gel G as the adsorbent and hexane-diethyl ether-formic acid (80-20-1) as the solvent. The fractions were detected under ultraviolet light (320 nm) after spraying the TLC plates with a methanolic solution (0.2%) of 2′7′-dichlorofluorescein, and the spots were detected using the standards for phospholipids, cholesterol, triacylglycerol, and cholesterol esters. Spots were scraped from the plates and collected separately in scintillation vials containing 5 ml of scintillation cocktail. The radioactivity of the samples was then measured using a liquid scintillation counter (LS-6500; Beckman Instruments, Fullerton, CA, USA).

### 2.5. Statistical Analyses

 All data were analyzed by analysis of variance using the GLM procedure of SAS (SAS Institute, Cary, NC). Individual treatment differences were tested by Duncan multiple-range test. Data were presented as means ± SDs. Differences were considered significant at the level of *P* < .05.

## 3. Results

 As shown in Table 1, dietary karaya saponin, *R. capsulatus *and their combination significantly reduced (*P* < .05) the hepatic total cholesterol and triacylglyceride levels whereas they increase bile acid concentration (*P* < .05). On the other hand, the fecal excretions of cholesterol and triacylglycerides were significantly increased by karaya saponin, *R. capsulatus *and their combination. The excretion of fecal bile acid was significantly higher for all the treatments (*P* < .05). The concentrations of total cholesterol and triacylglycerol in the serum and egg yolk were significantly decreased (*P* < .05) by karaya saponin, *R. capsulatus *and their combination. Serum cholesterol concentration was significantly decreased (*P* < .05) and serum HDL-cholesterol concentration was significantly increased (*P* < .05) in the karaya saponin and karaya saponin +  *R. capsulatus *groups compared with those in control group. 

 The effects of dietary karaya saponin and *R. capsulatus *on fatty acid composition in the egg yolk of laying hens are shown in Table 2. The concentrations of oleic, linoleic and linolenic acids in the egg yolk were significantly (*P* < .05) increased by the karaya saponin, *R. capsulatus *and karaya saponin +  *R. capsulatus *supplemented diets as compared with those with the control diet. Consequently, concentrations of polyunsaturated fatty acids (PUFA) were significantly (*P* < .05) increased in the egg yolk of karaya saponin, *R. capsulatus *and karaya saponin +  *R. capsulatus* supplemented groups compared with those of the control group. The ratio of PUFA to saturated fatty acids (SFA), and PUFA and monounsaturated fatty acids (MUFA) to SFA, in the egg yolk of the karaya saponin and karaya saponin +  *R. capsulatus *groups were significantly higher (*P* < .05) than those in the control group. 

**Figure 1:**
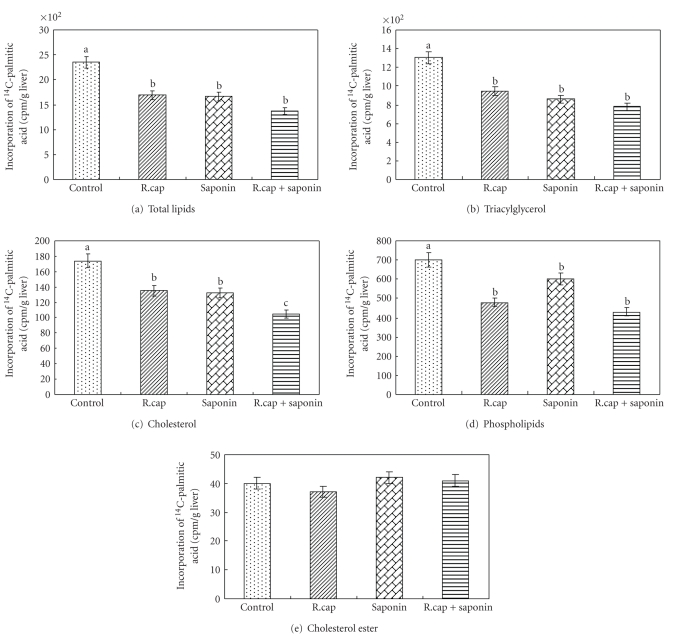
Effect of dietary karaya saponin and *R. capsulatus* on the incorporation of 1-^14^C-palmitic acid into hepatic (a) total lipids, (b) triacylglycerol, (c) cholesterol, (d) phospholipids, and (e) cholesterol ester fraction. Differences were tested by Duncan multiple-range test. ^a-c^Values with different superscripts differ significantly (*P* < .05); “a”, “b”, “c” indicate significant difference from each other. Values are mean ± SD, *n* = 10 laying hens.

 The effects of dietary karaya saponin and *R. capsulatus *on the incorporation of 1-^14^C-palmitic acid into hepatic lipids of laying hens are shown in Figure 1. There was a significantly lower (*P* < .05) incorporation of ^14^C into hepatic total lipids, triacylglycerides, and total cholesterol of hens in the karaya saponin, *R. capsulatus* and karaya saponin +  *R. capsulatus* supplemented groups than that in the control group. There was no significant difference in the incorporation of ^14^C from palmitic acid into the cholesterol ester fraction between groups. In the phospholipid fraction, all the treatments significantly reduced incorporation, and the trends of *R. capsulatus* and saponin +  *R. capsulatus* treatments were similar.

## 4. Discussion

 The present study expands our understanding of the cholesterol-lowering effects of karaya saponin and *R. capsulatus *in laying hens. In this study, we found that the fecal excretion of bile acids in the laying hens was significantly increased by karaya saponin, *R. capsulatus *or karaya saponin +  *R. capsulatus* supplemented diet compared with that with the control diet. Similarly, liver bile acid was also increased by all the treatments. In both feces and liver, the trends for bile acid were similar and the highest bile acid concentration was found when karaya saponin and *R. capsulatus*, were used in combination. This indicates that these two natural ingredients can function synergistically, at least in laying hens. These results are in agreement with our previous observations described in Afrose et al. [10] and Salma et al. [6]. Many pathways and factors have been reported to contribute to the hypocholesterolemic effects of different dietary saponins. Cholesterol reduction by karaya saponin and *R. capsulatus *is a new approach that continues to stimulate much research and discussion. Plant saponins exhibit many of the characteristics desirable for long-term hyperlipidemic therapy and thus may represent a novel form of therapy for the treatment of hypercholesterolemia [19]. In spite of the fact that a great variety of investigations on the biological activities of saponins have been carried out [20], studies on their effects on bile acid, as described here, are rare, aside from a number with steroid saponins. In this study, the consequent increase in fecal excretion is compensated for by enhanced hepatic conversion of cholesterol to bile acid. Support of this finding comes from an observation by Oakenfull and Fenwick [21] that plant fiber preparations containing saponins bind to bile acids in vitro. Oakenfull et al. [22] further concluded that 1% soyasaponins significantly increase fecal bile acid and neutral sterol excretion compared with a control diet without soyasaponin. From this study, it was also observed that cholesterol reduction and fecal cholesterol excretion are inversely correlated with similar magnitudes. Thus, it is conceivable from this study that the cholesterol-lowering effect of saponin might be due to the increase in bile acid excretion [23] that occurs through interference with the absorption of cholesterol [24]. Potter [25] also indicated that saponin might alter the absorption of cholesterol and bile acid. One of the possible mechanisms for this effect is that saponin forms micelles with bile acid [26]. It has been postulated that a saponin from platycodins may interrupt the formation of cholesterol micelles by cocrystallizing with cholesterol [27]. Lin et al. [28] also pointed out that saponin binds a certain amount of bile acid when added to a diet in rats. 

 The resulting decrease in cholesterol synthesis, which was observed in laying hens that were administered feed with karaya saponin and *R*. *capsulatus*, leads to hypocholesterolemia. The determination of the incorporation of labeled palmitic acid into total lipids and the various lipid fractions provides an indication of the pattern of lipid synthesis influenced by the combination of karaya saponin and *R*. *capsulatus *supplementation. However, our study indicates that the classes of liver lipid were not all affected by saponin or *R*. *capsulatus *in the same manner and magnitude. Decreased hepatic synthesis of cholesterol from precursor palmitic acid from the saponin or *R*. *capsulatus *supplemented diet is the most detectable hypocholesterolemic effect. Although the suppression of individual cholesterol synthesis was similar for individual saponin or *R*. *capsulatus*, the combination of these two caused a marked decrease. Thus, the synergistic effect of karaya saponin and *R. capsulatus *on the suppression of cholesterogenesis is in agreement with the low hepatic cholesterol concentration (as shown in Table 1). It was suggested that by virtue of their purported intestinal action, saponins could provide a nonsystemic alternative to the commonly used HMG-CoA reductase inhibitors [29]. It is possible that karaya saponin together with *R. capsulatus *suppresses cholesterol synthesis by reducing HMG-CoA activity. When palmitic acid is used as a substrate for lipid synthesis under *in vitro *conditions, dietary saponin and *R. capsulatus *could tend to reduce palmitic acid incorporation into some lipid classes by causing a reduction in malonyl-CoA formation. This suppression of cholesterol synthesis is in line with our previous observations in chickens [6, 10]. The pattern of palmitic acid distribution in the various lipid fractions of liver was different between the treatment group and the control. Synthesis of triacylglycerol predominates in the chicken liver, while karaya saponin and *R*. *capsulatus *markedly reduced this synthesis. When karaya saponin with *R. capsulatus *was fed to hens, there was an approximately 25% reduction in cholesterol synthesis. Thus, the suppressed esterification of 1-^14^C-palmitic acid into triacylglycerol and cholesterol is one of the potential hypocholesterolemic mechanisms of karaya saponin and *R. capsulatus *in laying hens.

 Simultaneously, the excretion of cholesterol through feces was also enhanced by the combined supplementation of karaya saponin and *R*. *capsulatus. *The evidence from this study suggests that this aspect of cholesterol trafficking is not the cause of cholesterol modulation. Using a single cholesterol tracer method, previously validated against the dual isotope ratio methodology, Southon et al. [2] have shown that cholesterol absorption increased in the animals receiving a high dose of saponin. Cholesterol excretion and hepato-biliary-intestinal efflux of cholesterol appear to be the mechanisms responsible for the lower circulating levels. However, our data suggest that karaya saponin acting synergistically with *R. capsulatus* likely increased fecal cholesterol excretion as fecal cholesterol concentration was increased 2-fold. In a recent study, it was observed that saponin enhanced intestinal sterol permeability, resulting in increased cholesterol absorption; on the other hand, there are still certain unknown mechanisms that exist that can enhance cholesterol efflux, resulting in a net depletion of the sterol pool [13]. It is conceivable from our study that hepatic increased bile acid resulted in fecal cholesterol excretion. The concentration of excreted cholesterol was 35% higher in the laying hens fed both the karaya saponin and *R. capsulatus *supplemented diet than in those fed the control diet. 

In this study, some fatty acids in egg yolk were altered by the karaya saponin and *R. capsulatus *supplementation. We previously reported that dietary supplementation of *R. capsulatus *altered the fatty acid composition of serum lipid in rats [5]. In the present study, the combination of dietary saponin and *R. capsulatus *resulted in a significant increase in the oleic, linoleic, and linolenic acid concentrations in egg yolk. In broiler chickens, Salma et al. [7] reported that the concentrations of oleic, linoleic, and linolenic acids in breast and thigh meat are markedly increased by the combined supplementation in chickens. Our findings clearly demonstrate that dietary *R. capsulatus *can improve the fatty acid composition in eggs. Although the levels of saturated palmitic and stearic acids were slightly increased by the *R. capsulatus *supplementation, they were markedly decreased when the combination of saponin and *R. capsulatus *was introduced to the diet. The alteration of the egg yolk fatty acid profile by saponin and *R. capsulatus *supplementation might provide health benefits. Despite the fatty acid alteration effects, nothing is known about the mode of action and mechanism by which karaya saponin enhances PUFA. Thus, it will be interesting to elucidate the mechanism underlying this phenomenon.

 Although it is difficult to completely characterize the mechanism of the action of karaya saponin, counteraction of bile activity might explain the reduced cholesterol bioaccessibility observed when karaya saponin is introduced into the diet. The mechanism of karaya saponin and *R. capsulatus *hypolipidemic action might also involve the posttranscriptional suppression of HMG-CoA reductase in a manner mimicking the action of putative nonsterol feedback inhibitors. Further studies are needed to confirm these effects. In conclusion, karaya saponin and *R. capsulatus *supplementation feeding resulted in a pronounced cholesterol-lowering effect. Our study suggests that the reductions in the levels of serum and egg cholesterol are caused by the suppression of cholesterol synthesis and the promotion of cholesterol catabolism in the liver.

## Figures and Tables

**Table 1:** Effect of dietary karaya saponin and *R. capsulatus *on LDL, HDL, total cholesterol, triacylglycerol, and bile acids in serum, liver, and feces of laying hens.

Parameter	Treatment			
	Control	*R. capsulatus*	Saponin	Saponin + *R. capsulatus *
Liver				
Cholesterol (mM/g)	1.10 ± 0.12^a^	0.59 ± 0.09^b^	0.61 ± 0.08^b^	0.47 ± 0.04^b^
Triacylglycerol (mM/g)	1.65 ± 0.15^a^	1.12 ± 0.10^b^	1.03 ± 0.04^b^	0.98 ± 0.03^b^
Bile acids (nM/g)	51.9 ± 3.25^a^	68.6 ± 3.16^b^	70.15 ± 4.22^b^	74.4 ± 3.35^b^
Fecal				
Cholesterol (mM/g)	0.71 ± 0.05^a^	0.96 ± 0.11^b^	0.98 ± 0.23^b^	1.20 ± 0.16^b^
Triacylglycerol (mM/g)	0.80 ± 0.17^a^	1.20 ± 0.21^b^	1.26 ± 0.14^b^	1.21 ± 0.12^b^
Bile acids (nM/g)	98.0 ± 5.35^a^	136.6 ± 6.23^b^	143.52 ± 5.54^b^	158.04 ± 7.15^c^
Serum				
Cholesterol (mM/L)	3.91 ± 1.18^a^	3.38 ± 0.42^b^	3.16 ± 0.32^b^	3.07 ± 0.45^b^
Triacylglycerol (mM/L)	4.58 ± 0.95^a^	4.26 ± 1.03^b^	4.29 ± 1.21^b^	4.05 ± 1.63^b^
LDL-cholesterol (mg/dL)	112.53 ± 3.44^a^	96.24 ± 3.26^ab^	91.67 ± 4.25^b^	80.16 ± 3.45^c^
HDL-cholesterol (mg/dL)	46.25 ± 2.36^a^	44.31 ± 4.13^ab^	50.33 ± 5.24^b^	58.81 ± 4.37^b^
Yolk				
Cholesterol (mM/g)	3.65 ± 0.89^a^	3.40 ± 0.74^b^	3.38 ± 0.55^b^	3.19 ± 0.62^b^
Triacylglycerol (mM/g)	23.6 ± 2.16^a^	20.7 ± 2.04^b^	21.71 ± 3.25^b^	19.86 ± 2.66^b^

^a − c^Values with different superscripts differ significantly (*P* < .05) in the same row; “a”, “b”, “c” indicate significant difference from each other while “ab” is not significant. Values are mean ± SD for 10 laying hens per group. Differences were tested by Duncan multiple range test. HDL = High density lipoprotein; LDL= Low density lipoprotein.

**Table 2:** Effect of dietary karaya saponin and *R. capsulatus* on yolk fatty acids.

Fatty acid	Treatment			
	Control	*R. capsulatus*	Saponin	Saponin + *R. capsulatus *
16 : 0	5.90 ± 0.48^a^	6.78 ± 0.99^ab^	5.83 ± 0.52^ab^	4.08 ± 0.65^b^
18 : 0	2.01 ± 0.19	2.81 ± 0.28	1.93 ± 0.17	1.85 ± 0.15
16 : 1	0.71 ± 0.07	1.08 ± 0.16	1.13 ± 0.20	1.11 ± 0.09
18 : 1	7.46 ± 0.88^a^	12.36 ± 1.14^b^	10.75 ± 1.84^b^	14.10 ± 1.65^c^
18 : 2	11.87 ± 0.97^a^	16.34 ± 1.97^b^	18.25 ± 2.01^b^	17.44 ± 1.88^b^
18 : 3	2.67 ± 0.17^a^	4.16 ± 0.53^b^	4.33 ± 0.62^b^	5.01 ± 0.41^b^
MUFA	8.10 ± 0.47^a^	12.89 ± 1.16^b^	11.75 ± 1.13^ab^	14.78 ± 1.23^c^
PUFA	14.47 ± 1.05^a^	20.50 ± 0.86^b^	22.16 ± 1.57^b^	22.08 ± 2.11^b^
SFA	7.89 ± 0.82	9.59 ± 0.54	7.48 ± 0.43	5.77 ± 0.22
PUFA : SFA	1.81 ± 0.09^a^	2.14 ± 0.08^ab^	2.91 ± 0.05^b^	3.80 ± 0.25^b^
(PUFA+MUFA) : SFA	2.82 ± 0.11^a^	3.25 ± 0.24^ab^	4.50 ± 0.31^b^	6.12 ± 0.35^b^

^a − c^Values with different superscripts differ significantly (*P* < .05) in the same row; “a”, “b”, “c” indicate significant difference from each other while “ab” is not significant. Values are means ± SD (mg/g) for 10 laying hens per group.

Differences were tested by Duncan multiple range test. MUFA = monounsaturated fatty acids, PUSA = polyunsaturated fatty acids, SFA = saturated fatty acids.
